# Extending Marine Species Distribution Maps Using Non-Traditional Sources

**DOI:** 10.3897/BDJ.3.e4900

**Published:** 2015-04-17

**Authors:** John Stephen Wood, Fabio Moretzsohn, James Gibeaut

**Affiliations:** ‡ Harte Research Institute, Texas A&M University-Corpus Christi, Corpus Christi, Texas, United States of America; § Harte Research Institute, Texas A&M University-Corpus Christi, Corpus Christi, Texas, United States of America

**Keywords:** Species, Distribution, Crowdsource, IUCN, Red List, Protocol, Geographic Information Systems, GIS, Biodiversity Databases, citizen science

## Abstract

**Background:**

Traditional sources of species occurrence data such as peer-reviewed journal articles and museum-curated collections are included in species databases after rigorous review by species experts and evaluators. The distribution maps created in this process are an important component of species survival evaluations, and are used to adapt, extend and sometimes contract polygons used in the distribution mapping process.

**New information:**

During an IUCN Red List Gulf of Mexico Fishes Assessment Workshop held at The Harte Research Institute for Gulf of Mexico Studies, a session included an open discussion on the topic of including other sources of species occurrence data. During the last decade, advances in portable electronic devices and applications enable 'citizen scientists' to record images, location and data about species sightings, and submit that data to larger species databases. These applications typically generate point data. Attendees of the workshop expressed an interest in how that data could be incorporated into existing datasets, how best to ascertain the quality and value of that data, and what other alternate data sources are available. This paper addresses those issues, and provides recommendations to ensure quality data use.

## Introduction

“How can we standardize the methods used to incorporate point data into distribution range polygons? How can we accelerate the collection of observation (point) data”? These questions were posed to an international group of taxonomists during a workshop that was held in conjunction with the IUCN Red List Gulf of Mexico Fishes Assessment Workshop, which was held at the Harte Research Institute for Gulf of Mexico Studies on the campus of Texas A&M University-Corpus Christi in January 2014. Table 1 lists attendees and their affiliations. This paper seeks to review the discussions that occurred during the workshop, to review existing protocols and to suggest possible changes that might enhance the current protocol and encourage inclusion of new and different data streams.

The goal of this workshop was to discuss a methodology for community-based recording of observations of marine species. These data, if collected in a repeatable and consistent manner over a long period of time, will become a valuable reference for distribution mapping for marine species ranges (adapted from Insley et al. (2008)). During this four-hour workshop several topics were discussed, including current Red List mapping standards, Biodiversity of the Gulf of Mexico (BioGoMx) database (Moretzsohn et al. 2011), species data and maps, and combining different types of geographic data, such as range maps, museum specimen records, field observations and other point data. A need for accelerating the collection and acceptance of point data, and facilitating expert review of species distribution and ranges is evident. Inclusion of alternate sources of data, such as fisheries data and that generated with crowdsourced applications was discussed. Ensuring the quality of new point data from a variety of sources was discussed, and should include considering how close to current range data the new points lay, how current the data is, and the possibility of establishing an ‘Index of Reliability’. The importance of including the costs of curating the specimens, spatial and attribute data in grants and funding requests was recognized. An additional topic of discussion during the workshop was whether the Red List spatial data can be useful in response to catastrophic events, such as the Deepwater Horizon oil spill in 2010, and what tools would need to be developed to do so. Discussion evolved around the ability to map species data for a given region using an online mapping portal, and to perform advanced spatial analysis on species distribution polygons and intersecting data sets. The purpose of these distribution polygons is to provide the current known distribution of the taxon within its native range (IUCN 2013), and the spatial information is subject to change between update intervals, usually at regional evaluation workshop intervals, and publication. The limits of distribution are determined using known occurrences of the taxon, and knowledge of its habitat preferences, remaining suitable habitat, elevation (depth) limits. Other parameters should be considered, such as time periods or seasons when a species might aggregate in a particular region, the effects of trophic structure, migration patterns and known threats. Other caveats include that, although the 2013 IUCN (Global) Red List contains assessments for more than 70,000 species, spatial data exists for only about 43,000 species, leaving 27,000 that would be omitted from a spatial query that was dependent upon them (IUCN 2014b). Of this total of 70,000 species, the IUCN Global Marine Species Assessment, led by the Species Survival Commission, estimates 20,000 species are classified as 'marine' (IUCN 2015). Participants discussed the inclusion of additional ‘crowd-sourced’ point-based observation data. In addition, participants suggested that habitats and habitat preferences should be considered in mapping ranges. Specifically, depth range intervals for certain species or species groups should be considered and noted. Mating or spawning aggregations, nesting areas, nursery habitats, areas of particular abundance, and areas of habitat preference should be considered when mapping. Areas which are vulnerable to threats, such as coral reefs or Essential Fish Habitats should be included in mapping efforts. These considerations are included in the existing IUCN mapping and evaluation protocols, which are discussed below.

## Current IUCN Mapping Protocols

**Figure 1.**
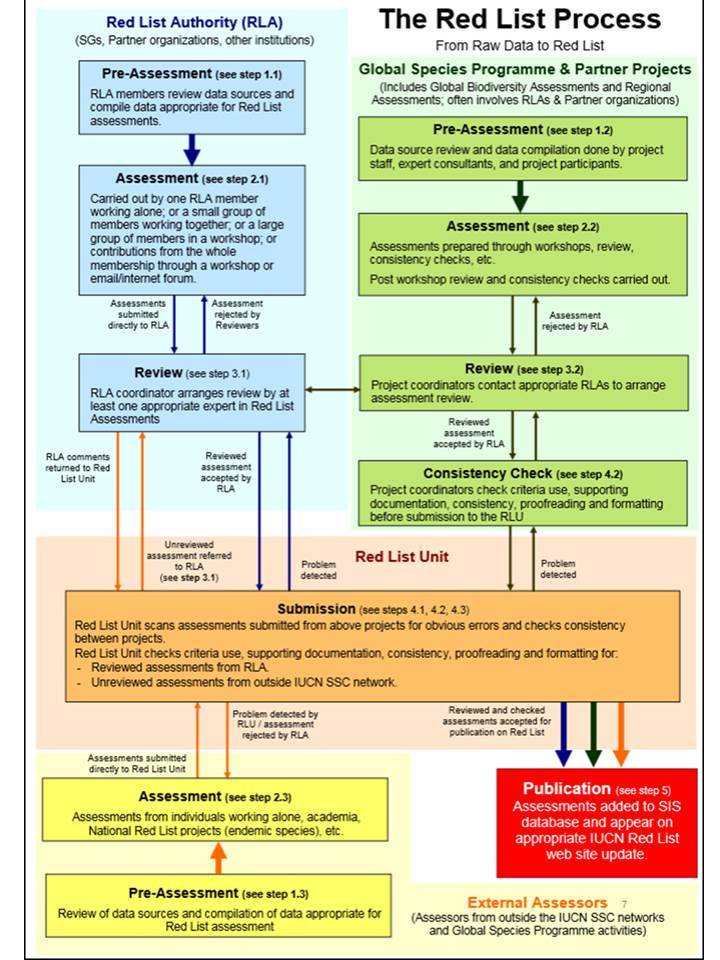
**The IUCN Red List Review Process** (IUCN 2014a). Steps refer to the DOCUMENTATION STANDARDS AND CONSISTENCY CHECKS FOR IUCN RED LIST ASSESSMENTS AND SPECIES ACCOUNTS (IUCN 2013).

The IUCN Red List of Threatened Species™ is essentially a checklist of taxa that have undergone an extinction risk assessment using the IUCN Red List Categories and Criteria, as shown in  Fig. 1. International Union for Conservation of Nature and Natural Resources (IUCN) and Global Marine Species Assessment (GMSA) have adopted the **Documentation Standards and Consistency Checks for IUCN Red List Assessments and Species Accounts** (IUCN 2013). These standards state that a distribution map is required as supporting information for all IUCN Red List assessments. The purpose of a distribution map is to provide the current known distribution of the taxon within its native range (IUCN 2013). The limits of distribution are determined using known occurrences of the taxon and knowledge of its habitat preferences, remaining suitable habitat, and elevation or depth limits. Further, it stipulates that a GIS shapefile (for geo-referenced polygons or point localities) is the preferable format for spatial data. Using shapefiles, an esri-developed geospatial vector data format, ensures their value for spatial data analyses, visual displays, and future functionality of the Red List website. IUCN gratefully acknowledges support from esri (Environmental Systems Research Institute) by providing GIS software and training under its Conservation Grant Program. If for some reason a shapefile cannot be created, other formats, including paper maps, text file coordinates, a pdf document, or a graphics file are also acceptable. This document also specifies a Red List standardized set of required attribute fields (Table 2) for shapefiles that will help maintain consistency within the IUCN database. Coded values for presence, origin and seasonality attributes may be found in the appendices of this document. The use of codes (see Tables 3, 4, 5) and subtypes eliminates synonyms, misspellings, and errors, and ensures that the data conforms to the schema of the Red List Species Information System (SIS) database.

The majority of assessments appearing on the IUCN Red List are carried out by members of the IUCN Species Survival Commission (SSC), appointed Red List Authorities (RLAs), Red List Partners, or participants of IUCN-led assessment projects (IUCN 2014a). However, assessments can be done by anyone and submitted to IUCN for consideration. The panels of species and taxonomic experts and authorities at IUCN workshops should be familiar with current IUCN mapping protocols, and also review Critical Habitat and Essential Fish Habitat mapping. The Guidelines for Appropriate Uses of Red List Data specifies that the maps that accompany many Red List Assessments are generalized, and should be used primarily as an orientation tool that represents the best data available at the time of review, not necessarily at time of publication, which can differ by several years. Environmental and threat level changes should also be reflected in the map review processes, as habitat conditions become either less or more desirable. The creation of Marine Protected Areas and protection of Essential Fish Habitats serve as prime examples of positive changes in habitat conditions. Negative changes such as major oil spills or hypoxic areas should also be reflected in the map review process. Changes in sea surface temperature should also be considered (Karnauskas et al. 2013). Current biotic and abiotic factors should be considered, and maps adjusted accordingly.

A detailed guidance document ‘Documentation standards and consistency checks for IUCN Red List assessments and species accounts’ (IUCN 2013) provides IUCN mapping standards for creating distribution maps to support Red List assessments. These distribution maps can be useful for identifying priority conservation areas, identifying gaps in scientific knowledge, and informing management decisions. A tutorial module is also available through the online IUCN Red List Training course **‘Module 5: IUCN Red List Mapping Standards’** (ConservationTraining 2013). This online tutorial takes approximately 1 hour to complete, and focuses on understanding the standards in place for creating species distribution maps for supporting IUCN Red List Assessments. Additional objectives for this tutorial are to aid in understanding the benefits of distribution mapping, recognize what information is being represented in the map, and know what mapping tools are available. Essential IUCN terminology, such as Extent of Occurrence (EOO) and Area of Occupancy (AOO) are explained.

Extent of Occurrence (EOO) is defined as the area contained within the shortest continuous imaginary boundary which can be drawn to encompass all the known, inferred or projected sites of present occurrence of a taxon, excluding cases of vagrancy (species far out of their typical range). This measure may exclude discontinuities or disjunctions within the overall distributions of taxa (e.g., large areas of obviously unsuitable habitat; but see 'area of occupancy'). Extent of occurrence can often be measured by a minimum convex polygon (the smallest polygon in which no internal angle exceeds 180 degrees and which contains all the sites of occurrence).

Area of Occupancy (AOO) is defined as the area within its 'Extent of Occurrence' (see definition above) which is occupied by a taxon, excluding cases of vagrancy. The measure reflects the fact that a taxon will not usually occur throughout the area of its extent of occurrence, which may, for example, contain unsuitable habitats. The area of occupancy is the smallest area essential at any stage to the survival of existing populations of a taxon (e.g. colonial nesting sites, feeding sites for migratory taxa). The size of the area of occupancy will be a function of the scale at which it is measured, and should be at a scale appropriate to relevant biological aspects of the taxon. The criteria include values in km^2^, and thus to avoid errors in classification, the area of occupancy should be measured on grid squares (or equivalents) which are sufficiently small.

The definitions above are taken directly from: http://www.iucnredlist.org/static/categories_criteria_2_3#definitions.

Distribution maps display a polygon intended to communicate that a species probably only occurs within its extent, which is based on known occurrences, knowledge of habitat preferences, remaining suitable habitats, elevation (or depth) limitations, and other expert knowledge. Point data, which can include line-based data from transects, polygon data from a defined area, such as a national park, and grid data (observations or survey records from a regular grid) from which these polygons are derived is obtained from published peer-reviewed literature, ‘grey’ literature (academic or government literature that is not formally published), field observations, biodiversity and taxonomic databases such as the Global Biodiversity Information Facility (GBIF) and Ocean Biodiversity Information System (OBIS), museum and other curated collections, or from taxonomic expert knowledge. There is a wide variety in the quality and quantity of these data. There are also online utilities, such as GeoCAT (Bachman et al. 2011) (http://geocat.kew.org/) which will display point data from a limited variety of sources. It is also capable of importing user-supplied point data. GeoCAT will calculate and display the EOO and/or AOO, and allow the selection and download of point data from a selected region or area.

The existing IUCN Red List mapping protocol for marine species differs from that for terrestrial species, primarily in that bathymetry may be used to delineate species range limits, much like elevation limitations may be used to limit the ranges of terrestrial species. It also differs from the mapping protocol for freshwater fishes, where drainage basins are typically used for determining and delineating range extents. The IUCN protocol for converting marine observation point data into distribution polygons involves a three-step process: Step One: Plot Observation Points, Step Two: Expand the Range, and Step Three: Refine the Range.

In Step One, point observation data are plotted. Since data often come from a diverse range of formats and sources, methods for plotting data points will vary. All data should be plotted in the Geographic Coordinate System, WGS-1984.

In Step Two of this protocol, the range is extrapolated based on the extent of suitable habitat (ESH) in the area and expert knowledge of the species and its requirements. Surrounding areas of similar habitat may be included. For terrestrial species, there are various other factors such as elevation, temperature, and even natural physical barriers, such as oceans. Marine species range may be affected by depth, water temperature gradients, salinity ranges, photic zone depths, and O_2_ concentrations. Often, this extrapolation is accomplished by buffering the point data, and then creating a convex polygon that surrounds the available points.

In Step Three, areas that are deemed unsuitable for a species are removed from the extrapolated habitat polygon(s). Note that this extrapolation and elimination of areas may result in discontinuous or non-contiguous polygons. This may result in different Extent of Occurrence and Area of Occupancy, and results in the best representation of the species’ likely occurrence or distribution based on currently available information. The Area Of Occupancy (AOO) will reflect influences from both biotic and abiotic factors.

A ‘Best Practices’ section of the tutorial offers several ‘rules of thumb’ to go by:

- Always name the polygon shapefiles by the taxon’s scientific name (using “genus_species” format)
- Smooth all polygons and check for irregularities before submitting
- Provide GIS data in geographic coordinates (specifically WGS84, the default setting for most GPS units)
- Remember that data attributes are absolutely required with spatial data, including codes for presence, origin and seasonality.

The distribution map produced with this protocol represents the taxon’s distribution within its overall range for communication and/or conservation planning purposes; it may not equate to either the spread of extinction risk (Extent Of Occurrence) or the occupied range area (Area Of Occupancy) as defined by the IUCN Red List Categories and Criteria, but can be used to support these measurements.

There are several areas where questions may exist that this protocol doesn’t address such as: what point data sources should be included, what areas should be eliminated, how should seasonality by represented (separate GIS file, separate polygon within the same GIS file…).

## Crowdsourcing Biodiversity Data

Much of the workshop discussion focused on bringing in additional data and data sources. With the advent of numerous portable electronic devices, including Smartphones, with different applications and interfaces and GPS/mapping capabilities, new and exciting sources of species and species/location data are available, which could be included with current datasets. Crowdsourcing, commonly known as ‘citizen science’, is a manner of collecting data and observations in which collaborators who may lack credentials and formal institutional affiliation can contribute to the work of taxonomists and scientists. For example, rather than requiring a master’s degree in ichthyology, a citizen science project might ask if a candidate can learn to identify a particular species of fish using a dichotomous key (Lauro et al. 2014). There are a variety of field guides and keys available for Smartphones, particularly for Android and iPhone systems. For example, the Audubon Society puts nature at your fingertips with fieldguides to butterflies, birds, trees, wildflowers, mammals, fishes, mushrooms, insects and spiders. In all, approximately twenty Audubon ‘apps’ are available at http://www.greenmountaindigital.com/audubon-guides.html.

The Field Studies Council and Burkmar, R. 2014) has published a paper discussing the changes in biological identification brought on by the rapid develoment of information technology. An entire section is devoted to decribing the various types of keys and field guides used for biologic identification.

To illustrate crowdsourcing, consider several examples:

- An enterprising PhD student, Devin Bloom, from the University of Toronto Scarborough successfully used FaceBook to post images and identify almost 5,000 fish specimens collected during the first ichthyologic survey on Guyana’s Cuyuni River. This feat was accomplished in less than 24 hours, by a network of friends, many of whom had PhD’s in ichthyology. The National Museum of Natural History and the Smithsonian Institute (Anonymous 2011) ran stories on the team’s novel use of social networking to crowdsource their data. FaceBook featured this as a “FaceBook Story of the Week”.  Bloom says “Social networking is so powerful, and scientists should be using it more to connect with the world-at-large”.  Bloom credits an ichthyologist at Texas A&M University, Nathan Lujan, who has been using FaceBook to assist in fish identification for several years, and introduced Bloom to the idea. https://www.facebook.com/facebook/posts/110150795733096.
- Murthy et al. (2013) developed an application (**SuperIDR**) for tablet computers for fresh-water fish identification. A user may annotate fish images and identify fish from Virginia using a dichotomous key. Students using this software demonstrated an enhanced ability to correctly identify fish species. Crowdsourcing the species identification is specifically mentioned as a possibility. Because the software uses a downloadable digital library of images, the application can be useful as a field identification tool without an online connection. Because it is open-sourced, this application is expandable to serve other regions.
- ReefID.org (2015) offers an iPhone 'app' that encourages the user to upload images of reef fish for identification assistance by its members. They are collecting imaes from both amatuer and professional underwater photographers, in an effort to create the largest online collection in a user-friendly database. The app also has information on local (Caribbean) lodging and amenities, snorkeling/diving opportunities, land-based activities, restaurants and bars. The site also has information on how to turn your cell phone into an underwater camera, as well as how to add light to underwater shots. An Android version is in the beta stages.
- Dr. Amanda Vincent, Director of *Project Seahorse*, a joint marine conservation initiative with the University of British Columbia and Zoological Society of London, worked with the John G. Shedd Aquarium to develop the **iSeaHorse Explorer** (http://iseahorse.org/?q=about) (Project iSeahorse 2014, an ‘app’ that allows citizen scientists to contribute images and location data to the knowledge base on seahorses, pipefish and sticklebacks. Of the 48 seahorse species listed on the IUCN Red List of Threatened Species, 26 are considered ‘Data Deficient’; there isn’t enough information to determine the status and trends of their populations. This single app may expand the numbers of people collecting distribution data on seahorses from a handful to potentially thousands.
- Perhaps even more telling: an ‘app’ called ‘**Sea Turtle Tracker Tool**’ for Android-based Smartphones (http://alturl.com/9eedu) Chiral Labs (2011) was ‘developed in less than 48 hours’. This self-admitted ‘crude, rapid prototype’ was inspired by the work of the Marine Turtle Assessments program of the NOAA Fisheries Office in Hawaii. The ‘Sea Turtle Tracker’ app, with a file size of 593 kb, installs in less than 10 seconds, and allows the user to submit a photo-log, presumably to the International Sea Turtle Observation Registry (iSTOR), complete with GPS coordinates, time/date information, and a general description. A link on the app interface takes the user to www.seaturtle.org for more information.
- **MedMIS** is an ‘app’ developed by Geographica for monitoring and reporting invasive non-native species in Marine Protected Areas in the Mediterranean Sea. This app is based on a recent IUCN publication and contains information on major marine invasive species (Otero et al. 2013).
- The **iNaturalist** app for iPhones and Android smartphones encourages the citizen scientist to explore the natural world, learn about life, and record and add their own observations.  To report an occurrence, the user takes an image using a smartphone, enters or searches for a species name, selects an ongoing project if one exists, requests an identification from ‘the community’, and chooses to either display, generalize or hide the coordinates (for privacy issues). This information is then synchronized (‘Sync’) with the online account. The online interface allows a user to click to enter a location. Other fields can be added by the user, to meet criteria of certain projects, etc. A member can compile a ‘Life List’, and batch-load observations (up to 100 at a time) and images from cloud storage. This site has a Data Quality Assessment section, with categories for: Community Supported ID? Date? Georeferenced? Photos or sound? Wild /naturalized? Does the location seem accurate, does the date seem accurate? Is the entry appropriate? All observations receive a data quality assessment of ‘casual’, and achieve ‘research grade’ when certain conditions are met. Observations can also be degraded to ‘casual’ if certain conditions aren’t met or ‘the community’ of registered users agrees that the data ‘doesn’t look accurate’.

This small sampling of crowd-sourced data collection applications emphasizes the need to achieve a consensus on whether information collected in this manner can be used to enhance the current point and polygon observation data used to determine range and distribution extent information.

Crowd-sourced data using some of the ‘apps’ mentioned above would require a minimum of data fields be filled; other attributes should be added from existing and expert knowledge or specimen voucher information. The scientific name (binomial), the name of the compiler or submitter, and the citation (organization or app name) should be collected and added to the geo-tagged image information when available. Many of the apps and website entry points currently available fail to generate useable data, because they do not conform to taxonomic standards, or lack georeferencing. Database curators and developers now have access to several 'toolkits', such as that available from Indicia (2015) that will ensure that data collected conforms to standards such as the World Register of Marine Species (WoRMS). Compiled data sets should have appropriate metadata, formatted to international standards such as the Darwin Core. GBIF has an online tool, Darwin Core Archive Validator, as an aid to the validation process.

The Museum of Vertebrate Zoology (MVZ) at Berkley publishes a guide (Anonymous 2005) that describes and gives examples of a complete set of locality information, including a descriptive locality, to be used even when geographic coordinates are available. Elevation data, coordinates, datum, GPS accuracy, extent, references including a map name or GPS model, and a source for elevation data are required. Shah (2014) discovered that certain fields, such as *scientificName*, *recordedBy*, *eventDate*, *country* and *stateProvince* were more commonly recorded correctly than others such as *scientificNameAuthorship* and *verbatimLocation*, *county* and *locality*, which might be misunderstood or confused. The use of drop-down field values and auto-completing text enhances the accuracy of crowd-sourcing information, according to Shah (2014). It is possible that an app could be adapted or developed to require the minimal data fields be filled in for submission, and employ drop-down fields and autocomplete functions to eliminate a certain amount of error. For example, projects in iNaturalist can set some fields as required, limit the geographic distribution to a certain area, or the taxonomic scope to one taxon, before new observation can be submitted. Submissions should still be evaluated by regional and/or global evaluation teams, who would determine if the data is usable. Table 2 shows the fields required for an IUCN-accepted shapefile; automated filling of many of these fields should be programmed into a data collection app. When accumulted points are assembled into a single shapefile, other fields (such as 'compiler') should be completed. Shi  offers a list of Best Practices about Species Mapping that is also helpful (Shi 2011).

Chamberlain (2014) takes crowdsourcing to another level, by introducing 'groupsourcing', defined as: completing a task using a group of intrinsically motivated people of varying expertise connected through a social network. A 'group' is a feature of social networking in which a small set of users communicate through a shared message system, and perform one of three types of tasks.  Chamberlain defines the first of these tasks as a Task Request (TR): groups that encourage members to perform tasks *such as marine creature identification.* This paper goes on to discuss the accuracy of various methods, processing methodologies, ways of aggregating the collected data, and some of the limitations inherent in social networking and crowd/groupsourcing. Chamberlain concludes that groupsourcing offers a high accuracy compared to 'other methods of crowdsourcing', as well as being data-driven and low cost. Chamberlain also suggests that citizens, species experts and enthiusiasts can form these groups on social networking sites rather easily, which can allow citizen scientists to participate without much in the way of specialized equipment. Helping identify marine species in video and images from Remotely Operated Vehicles (ROVs) allows pareticipation without ever leaving your desk.

## Traditional Sources of Biodiversity Data

The traditional sources of biodiversity data include but certainly aren't limited to museum collections, taxonomic monographs, and biodiversity databases, which obtain much of their data from the first two sources. Individual specimen and observations within these collections come from a variety of sources, including published and unpublished (grey) literature, amatuer naturalists, and volunteer recorders (Issac et al. 2013). Among the largest biodiversity databases include the Global Biodiversity Information Facility (GBIF) with over 500 million data points, Ocean Biogeography Information System (OBIS) with over 40 million points of marine data, and HERPNET (soon to be replaced by VertNet), with over 14 million records of vertebrates. BioGoMx is a smaller but comprehensive database focused on the Gulf of Mexico, with over 15,000 species. Population distributions in BioGoMx are represented using an octant structure, dividing the Gulf of Mexico into eight octants. Each octant is further divided into six bathymetric depth ranges, thus resulting in a total of 48 polygons in the Gulf (Moretzsohn et al. 2011). The presence (or absence) of each species is determined for each octant (from published, peer-reviewed sources), and further discriminated into depth ranges, based on the depth distribution reported for each species by the taxonomic expert in the comprehensive biotic inventory of the Gulf of Mexico (Felder and Camp 2009). Integrated Digitized Biocollections (iDigBio) is digitizing information about existing, vouchered natural history collections, and is funded by the National Science Foundation as part of the Advanced Digitization of Biodiversity Collections (ADBC) program. This program has digitized over 24 million specimen records and over 4 million media records in 361 record sets. Images comprise only 286,000 of these records. Data and images for millions of biologic specimens are being made available in electronic format for the research community, government agencies, students, educators, and the general public. Approximately 4.5 million of these records are dated within the last 30 years. Kroupa and Remsen (2010) estimate that more than 100,000 scientific biological records, observations and specimens are recorded daily, through this initiative and others. They stress that the use of electronic tools and software can facilitate the recording of species datasets and minimize the number of errors, which can be within the geo-referencing domain as well as the taxonomic domain.

In addition to biodiversity databases, surveys, often conducted by state and federal fisheries agencies, are another source of biodiversity data. The Texas Parks and Wildlife Department has been conducting seine, gill net and trawl surveys since the 1970’s. The Louisiana Department of Wildlife and Fisheries has been collecting fishery independent data since 1988, from programs utilizing various gear and sampling techniques. The Florida Fisheries-Independent Monitoring program began the same year. The Southeast Area Monitoring and Assessment Program (SEAMAP) Gulf of Mexico component has been operational since 1981, planning, coordinating and conducting surveys for the Gulf States Marine Fisheries Commission. The Secretary of Agriculture in Mexico and the Instituto Nacional de Pesca (National Fisheries Institute) coordinate and conduct scientific and technological research on fisheries.

## Quality of Published Biodiversity Data

Biodiversity databases and literature contain vast amounts of distribution and taxonomic information, however the quality, scope  and scale of data varies. To address this potential problem, data should be verified and vetted by species experts and other knowledgeable workers before the information can be incorporated into Red List assessments. Taxonomic information can be verified in authoritative taxonomic databases such as the Integrated Taxonomic Information System (ITIS), the World Register of Marine Species (WoRMS), and other initiatives, which count on the assistance of taxonomic experts to keep the information as current as possible.

Biodiversity databases such as GBIF, OBIS, and Red List usually rely on multiple biodoversity and taxonomic databases to keep information current. It is recommended that any change in taxonomy be fully documented and linked to the source of the taxonomic authority. Similarly, information on potential mis-identifications should be provided to avoid potential problems.

Distributional data can have several sources of errors including incomplete or vague locality descriptions, wrong information from original source, transcription of data from hand-written labels and field log books, transposition of latitude and longitude, and GPS or other instrument error or calibration problems. Therefore, biodiversity databases should have fields for accuracy and data confidence, ideally reviewed by staff or an expert. If the data point is considered problematic, it should be flagged as such so that users can evaluate its usefulness. There are numerous database validation tools available.

## Conclusions and Recommendations

This paper is not intended to present all the possible combinations of crowdsourcing data or species evaluations, but instead should serve as a starting point for further discussion.

The review process:

- In addition to the attributes currently required for inclusion in the IUCN data base, spatial data on distribution during different life stages, seasonality, and depth ranges would be helpful in the evaluation and assessment process. Ancillary information such as competitor and predator expansion and invasions, disease ‘hotspots’, environmental and habitat degradation are vital to the distribution mapping and evaluation processes.
- The existing Red List mapping protocol for marine species, (Steps One, Two and Three as described briefly above), is sufficient for adding point data for inclusion into and redrawing existing species distribution polygons, if species experts and evaluators confirm the validity and accuracy of the data. Expert evaluation and acceptance are key to this process!
- Maps are produced and data collected for specific purposes, and should be used for other purposes only with extreme caution. Users should understand the purpose of a distribution map (is this map showing the possible range (EOO), or where the species is actually presumed to be limited to (AOO) by habitat and other factors), and the limitations inherent in that map.
- There are millions of data points of marine species currently available in biodiversity databases such as GBIF, OBIS, and others, which can complement and confirm distributions. Also, digitization of museum collection efforts such as iDigBio will produce millions of additional data points. Inclusion of these points also requires expert vetting. Experts should be aware of these possible sources, and relavant sites should be reviewed during the planning stages of the review process.

Crowd-sourced data:

- Crowdsource and ‘citizen science’ data can considerably increase the amount of data available for evaluating species distribution. Auto-collection of geo-locations, the use of autocomplete functions and drop-down lists can substantially add to the accuracy of that data.
- Crowdsourcing can be used to screen the majority of data on common species to reduce the workload of species experts, leaving the data on less common species or the observations outside of current polygons to be reviewed by experts.
- Points located outside of the existing polygons should be examined carefully.
- Points located inside existing distribution polygons may confirm the information is still current.
- Point sets should be examined in relation to the date of collection.
- In most cases, points are only an indication of a successful sampling effort. Points alone only indicate that a species was sighted at a given time and location. Lack of a point in a location does not preclude it being there.
- Further spatial analysis is often possible with mixed and 'noisey' data sets, using spatial and statistical analysis techniques. Inference of absence requires further analysis.
- New records of species not previously reported from an area could be a gap in knowledge, range extension due to introduction or climate change, or an error; prudence should be taken to verify the information with additional observations or rely on expert knowledge.
- Projects that count on ‘citizen scientist’ contributions to produce biodiversity data or to check data quality should be focused in scope and establish a minimum data quality standard; however, the required information should be as simple as possible to provide to avoid eliminating crowdsource data (i.e., if too much information required for contributions, the public may not get engaged).
- Crowdsourcing applications should include ample instructions, dropdown fields and auto-fill options where practical, leave little room for error, and strive for accuracy wherever possible. Data should be vetted by experts in the field of study.

## Figures and Tables

**Table 1.** Red List Workshop (Jan. 2014) Attendees

**First Name**	**Last Name**	**Affiliation**
Beth	Polidoro	IUCN/Arizona State University
Bruce	Collette	Smithsonian Institute/ chair of Tuna and Billfishes SSG
Christi	Linardich	IUCN/Old Dominion University
Fabio	Moretzsohn	Harte Research Institute
George	Sedberry	NOAA Office of National Marine Sanctuaries
Gina	Ralph	IUCN/Old Dominion University
Heather	Harwell	IUCN/Christopher Newport University
Hector	Espinosa-Perez	Instituto de Biología, UNAM, Mexico
Howard	Jelks	USGS Southeast Ecological Science Center
James	Tolan	Texas Parks and Wildlife Department, Coastal Fisheries Division
Jeff	Williams	Smithsonian Institute
Jim	Cowan*	Louisiana State University
John	McEachran	Texas A&M University
John	Wood	Harte Research Institute
Jorge	Brenner	The Nature Conservancy, Corpus Christi
Kathy	Goodin	NatureServe
Ken	Lindeman*	Florida Institute of Technology/Co-Chair of Snapper, Sea Bream, Grunt SSG
Kent	Carpenter	Old Dominion University/Manager IUCN Marine Biodiversity Unit
Kyle	Strongin	IUCN/Arizona State University
Labbish	Chao	Museum of Marine Biology & Aquarium, Taiwan/Sciaenidae SSG coordinator
Luiz	Rocha	California Academy of Sciences/ member of Groupers and Wrasses SSG
Luke	Tornabene	Texas A&M University
Maria	Vega Cendejas	CINVESTAV-IPN, Unidad Merida, Mexico
Mia	Comeros-Raynal	IUCN/Old Dominion University
Michelle	Zapp Sluis	Harte Research Institute
Riley	Pollom	Project Seahorse – University of British Columbia Fisheries Centre, Canada
Rodolfo	Claro	Instituto de Oceanología CITMA, La Habana, Cuba
Roger	McManus	IUCN, Arizona
Ross	Robertson	Smithsonian Tropical Research Institute, Panama
Tomas	Camarena Luhrs	National Commission of Natural Protected Areas–SEMARNAT, Mexico

**Table 2.** Required Attributes for IUCN Distribution Shapefiles (IUCN 2014b).

**Field**	**ESRI Field Type**	**Description**	**Required for Crowdsource Data**
ID_NO	Integer	Internal Record ID	Assigned by IUCN
BINOMIAL	String	Scientific name of the species	Recommended but not necessary
BASINID (for freshwater species only)	Integer	River Basin ID (Hydrosheds). (Note that this field is only included when species are mapped using the freshwater mapping protocol)	
PRESENCE	ShortInt	Is/Was the species in this area, codes listed below	Assigned by IUCN
ORIGIN	ShortInt	Why/ How the species is in this area, codes listed below	Assigned by IUCN
SEASONAL	ShortInt	What is the seasonal presence of the species in the area, codes listed below	Assigned by IUCN (by date/time stamp?)
COMPILER	String	Name of the individual/s or institution/s responsible for generating the polygon, if not IUCN.	Yes, with contact information (usually email address)
YEAR	ShortInt	Year in which the polygon was mapped, compiled, or modified	Date Field
CITATION	String	Individual/s or institution /s responsible for providing the data	Assigned by IUCN/app?
SOURCE	String	Source of distribution range given.	Yes (app name?)
DIST_COMM	String	Distribution comments that refer directly to the polygon.	Optional
ISLAND	String	Name of the island the polygon is on	Bay system or other geography?
SUBSPECIES	String	Epithet	Optional
SUBPOP	String	Epithet	Optional
TAX_COMM	String	Taxonomic comments that refer directly to the polygon. Includes notes on polygons pertaining to subspecies or subpopulations.	Assigned by IUCN
LEGEND	String	Code containing the combinations of the presence, origin and seasonality fields determining how the map will be displayed on The IUCN Red List website.	Assigned by IUCN

**Table 3.** Coded Domain Values for Presence (IUCN 2014b).

**Code**	**Presence**
1	Extant
2	Probably Extant (discontinued)
3	Possibly Extant
4	Possibly Extinct
5	Extinct (post 1500)
6	Presence Uncertain
**Description of Coded Values:** **Extant** – The species is known or thought very likely to occur presently in the area, which encompasses localities with current or recent (last 20-30 years) records where suitable habitat at appropriate altitudes remains. Extant ranges are included in the calculation of the extent of occurrence (EOO) and in maps of the historical distribution (See Note 5) of the species. **Probably Extant** – This code value has been discontinued for reasons of ambiguity. It may exist in the spatial data but will gradually be phased out. **Possibly Extant** – There is no record of the species in the area, but the species may possibly occur, based on the distribution of potentially suitable habitat at appropriate altitudes, although the area is beyond where the species is Extant (i.e., beyond the limits of known or likely records), and the degree of probability of the species occurring is lower (e.g., because the area is beyond a geographic barrier, or because the area represents a considerable extension beyond areas of known or probably occurrence). Identifying Possibly Extant areas is useful to flag areas where the taxon should be searched for. Possibly Extant ranges are not included in the calculation of EOO or in maps of the current and / or historical distribution of the taxon. **Possibly Extinct** – The species was formerly known or thought very likely to occur in the area (post 1500 AD), but it is most likely now extirpated from the area because habitat loss and/or other threats are thought likely to have extirpated the species, and there have been no confirmed recent records despite searches. Possibly Extinct ranges are not included in the calculation of EOO, but are included in maps of the historical distribution of the taxon. **Extinct** – The species was formerly known or thought very likely to occur in the area (post 1500 AD), but it has been confirmed that the species no longer occurs because exhaustive searches have failed to produce recent records, and the intensity and timing of threats could plausibly have extirpated the taxon. Extinct ranges are not included in the calculation of EOO, but are included in maps of the historical distribution of the taxon. **Presence Uncertain** – A record exists of the species' presence in the area, but this record requires verification or is rendered questionable owing to uncertainty over the identity or authenticity of the record, or the accuracy of the location. Presence uncertain records are not included in the calculation of EOO or in maps of the historical distribution of the taxon. **Notes**: 1. These codes are mutually exclusive, e.g. a polygon coded as “Extant” cannot also be coded as “Extinct”. 2. In accordance with the Red List Categories and Criteria, Extant polygons can include inferred or projected sites of present occurrence (see the Guidelines for Using the IUCN Red List Categories and Criteria for further guidance). 3. When there is uncertainty as to whether or not a species still occurs in an area in which it was formerly known to occur (usually because there have been no recent surveys), it is necessary for assessors to judge whether it is more appropriate to assign a coding of Extant or Possibly Extinct (based on available knowledge of remaining habitat, intensity of threats, adequacy of searches, and other evidence). 4. EOO calculations should be based on polygons coded as Extant only. 5. Maps of the historical range of a species can be produced by combining polygons coded as Extant, Probably Extant, Possibly Extinct, and Extinct. 6. The old Presence code 2 (Probably Extant) is now discontinued.	

**Table 4.** Coded Domain Values for Origin (IUCN 2014b).

**Code**	**Origin**
1	Native
2	Reintroduced
3	Introduced
4	Vagrant
5	Origin Uncertain
**Description of Coded Values:** **Native** – The species is/was native to the area **Reintroduced** - The species is/was reintroduced through either direct or indirect human activity. **Introduced** – The species is/was introduced outside of its historical distribution range through either direct or indirect human activity. **Vagrant** – The species is/was recorded once or sporadically, but it is known not to be native to the area. **Origin Uncertain** -The species’ provenance in an area is not known (it may be native, reintroduced or introduced) Note: These codes are mutually exclusive; a polygon coded as “Native” cannot also be coded as “Introduced”.	

**Table 5.** Coded Domain Values for Seasonality.

**Code**	**Seasonality**
1	Resident
2	Breeding Season
3	Non-breeding Season
4	Passage
5	Seasonal Occurrence Uncertain
**Description of Coded Values:** **Resident** – the species is/was known or thought very likely to be resident throughout the year **Breeding Season** – The species is/was known or thought very likely to occur regularly during the breeding season and to breed. **Non-breeding Season** – The species is/was known or thought very likely to occur regularly during the non-breeding season. In the Eurasian and North American contexts, this encompasses ‘winter’. **Passage** – The species is/was known or thought very likely to occur regularly during a relatively short period(s) of the year on migration between breeding and non-breeding ranges. **Seasonal Occurrence Uncertain** – The species is/was present, but it is not known if it is present during part or all of the year.	
